# Team care to cure adolescents with braces (avoiding low quality of life, pain and bad compliance): a case–control retrospective study. 2011 SOSORT Award winner

**DOI:** 10.1186/1748-7161-7-17

**Published:** 2012-09-20

**Authors:** Marta Tavernaro, Anna Pellegrini, Fabrizio Tessadri, Fabio Zaina, Andrea Zonta, Stefano Negrini

**Affiliations:** 1 ISICO (Italian Scientific Spine Institute), Trento, Italy; 2 Casa di Cura “Villa Regina,” Arco di Trento (TN), Trento, Italy; 3 Orthotecnica, Trento, Italy; 4 Ospedale di Bressanone, Bressanone, Italy; 5 ISICO (Italian Scientific Spine Institute), Milan, Italy; 6 University of Brescia, Brescia, Italy; 7 Fondazione Don Gnocchi, Milan, Italy

## Abstract

**Background:**

Bracing could be efficacious, given good compliance and quality of braces. Recently the SOSORT Brace Treatment Management Guidelines (SBTMG) have highlighted the perceived importance of the professional teams surrounding braced patients.

**Purpose:**

To verify the impact of a complete rehabilitation team in the adolescent patient with bracing.

**Materials and methods:**

Design. Initial cross-sectional study, followed by a retrospective case–control study. Population: Thirty-eight patients (15.8 ± 1.6 years; 26 females; 10 hyperkyphosis, 28 scoliosis of 29.2 ± 7.9° Cobb) extracted from a single orthotist database (between January 1, 2008 and September 1, 2009) and treated by the same physician; brace wearing at least 15 hours/day for a minimum of 6 months; age 10 or more. Treatment: Braces: Sforzesco, Sibilla, Lapadula or Maguelone. Exercises: SEAS. Methods: Two questionnaires filled in blindly by patients: SRS-22 and one especially developed and validated with 25 questions on adherence to treatment. Groups (main risk factor): TEAM (private institute: satisfied 44/44 SOSORT criteria; grade of teamwork, “excellent”) included 13 patients and NOT 25 (National Health Service Rehabilitation Department: 35/44 SOSORT criteria respected; grade, “insufficient”).

**Results:**

TEAM was more compliant to bracing than NOT (97 ± 6% vs. 80 ± 24%) and performed nearly double the exercises (38 ± 12 vs. 20 ± 13 minutes/session). The self-reduction of bracing was significant in NOT (from 16.8 ± 3.7 to 14.8 ± 4.9 hours/day, , P<0.05); TEAM showed a significant reduction in the difficulties due to bracing (from 8.9 ± 1.4 to 3.5 ± 2.0 in 12 months on a 10-point scale, P<0.05). Pain was perceived by 55% of NOT versus 7% of TEAM (P < 0.05). The populations did not differ at the baseline studied outcomes. The absence of a good team surrounding the patient increases by five times the risk of reduced compliance to bracing (odds ratio OR 5.5 – 95% confidence interval 95CI 3.6-7.4), along with more than 15 times that of QoL problems (OR 15.7 - 95CI 13.6-17.9) and pain (OR 16.8 - 95CI 14.5-19.1).

**Conclusions:**

Provided the limits of this first study on the topic, the SBTMG seems to be important for brace treatment, influencing pain, QoL and compliance (and so, presumably, final results). Future studies on the topic are advisable.

## Background

The recently published Cochrane Review on the efficacy of bracing for adolescent idiopathic scoliosis (AIS) concludes in favor of the efficacy of bracing, even if the quality of evidence is very low, and it is possible that future studies could change the actual results [1,2]. This is consistent with the doubts raised by a metanalysis of the English literature, which showed the same efficacy of bracing without exercises and watchful waiting [3]. In Europe, when exercises are added to bracing, the results are apparently different [1,2,4-8]. The studies following the SRS criteria for bracing on rigid TLSOs seem to confirm this hypothesis, with various results in the U.S. [9,10], good results in Europe [11,12], using also exercises [12].

The possible reasons reported in the literature for the low efficacy of bracing in some settings include quality of bracing and compliance. Studies [13,14] showed that immediate in-brace correction (as an estimate of brace quality) and compliance allow one to predict the final results of bracing. More recently, a predictive formula for final results has been developed using compliance (checked with heat sensors), the risk of progression at the start of bracing and the tightness of the brace closure [15].

Nevertheless, adherence to treatment and compliance are characteristic neither of treatment nor of the patient but come from the good interaction between these two factors. During the development of “Consensuses by the International Society on Orthopedic and Rehabilitation Treatment (SOSORT)” it quickly became clear that there was no agreement on the way to act on the spine to produce results [16] or on the braces to be used [17-20]. However, there was consensus among the internationally recognized experts on bracing regarding the importance of treatment management criteria. This motivated the development of the Clinical Guidelines Consensus on Brace Treatment Management [21] which reports recommendations on the following domains: Experience/competence, Behaviors, Prescription, Construction, Brace Check and Follow-up. The main concept behind those guidelines is the need for a multiprofessional expert team to effectively treat the patient through increased compliance. Even if this is the consensus by SOSORT experts, to date there is not a study documenting what can be the impact on patients of a multiprofessional team following the SOSORT criteria for brace management.

The purpose of this paper is to verify what could be the effect of a complete, expert rehabilitation team respecting the SOSORT criteria [21] compared to an incomplete team. Particularly, in this study we focused on the role of allied professionals (AP) (in our study, because of our Health System, they were physiotherapists, but in other Countries the same role could be covered by other professionals) beyond the technical aspect of the exercises proposed, as the everyday aggregator of the overall team.

## Materials and methods

### Design

Because this is the first research approach to this specific topic in the literature, prior to approaching toward future prospective controlled studies a retrospective case–control design was planned after an exploratory cross-sectional study.

### Population

The studied population has been extracted from the entire database of a single orthotist, including all his patients from January 1, 2008 to September 1, 2009. We considered only the patients treated by a single physician, and that physician worked at the same time in two completely different teams in the same Italian region, thereby allowing us to study the chosen risk factor.

The inclusion criteria were as shown below:

Adolescents (10 years or more): Able to answer autonomously to questionnaires;

Brace treatment for either idiopathic scoliosis or hyperkyphosis: We looked at the effect of the brace on adolescents and not on pathology;

At least 15 hours/day of brace wearing: To guarantee that there was still an interference of the brace with out-of-home daily activities;

Patients in brace since at least 6 months: To guarantee enough experience in brace wearing.

Out of a total of 360 braced patients in the orthotist database for the studied period we excluded: 264 because treated by other physicians, 55 because they were wearing the brace less than 15 hours/day or since less than 6 months, and 3 more because had less than 10 years of age. In the end, 38 patients fulfilled the inclusion criteria. We had 26 females and 12 males, age 15.8 ± 1.6 years. Of this number, 28 had idiopathic scoliosis that, at the start, was of 29.2 ± 7.9° Cobb but had been reduced during treatment of 6.5 ± 4.6° (P < 0.001). Patients had been braced since 15 ± 4 months, and the actual prescription included brace for 17.1 hours per day (range 15–23) and regular exercises. All patients were in treatment with one of the following braces: Sibilla or Sforzesco for idiopathic scoliosis, Maguelone for hyperkyphosis or Lapadula for hyperkyphosis and scoliosis. All patients performed SEAS exercises [7,12,22].

### Methods

Patients have been evaluated through two questionnaires: the SRS-22 (validated Italian version) and one specifically developed and validated (through pre-test and test-retest procedures) with 25 ordinal multiple choices, binary or numerical questions about adherence to treatment (sections: brace, exercises, team) Additional File ( 1). All questionnaires have been proposed blindly by a person not involved in the treatment. Patients filled in the two questionnaires at home. In order to preserve and guarantee that the answers could be totally anonymous, the completed questionnaires were posted by the participants in a closed box (one per setting involved) that was opened only after a specific period of time. Two reminders were sent to patients before opening the box and collecting the answers.

### The studied risk factor: team approach

The population was split into two groups according to the setting in which treatment has been performed. In fact, since the population was chosen as having been treated by the same orthotist and physician, the main (and only) distinction between the two populations was in the physiotherapeutic and general team approach.

The exercises proposed by both groups followed the SEAS school [7,12,22]. At the time when the study was performed, a certification in the SEAS approach did not exist. Nevertheless, all APs participated in a specific training in the SEAS approach, and they were all supervised by the same physician participating in the study. Today, the SEAS approach includes also the team and psychological approach to patients, but it was not specifically addressed during courses before the publication of the SOSORT Guidelines on bracing [21].

To check for the possible differences between the two team settings, the questionnaire proposed by SOSORT [21]http://www.scoliosisjournal.com/content/4/1/2/additional) have been used. Answers to the questionnaire were given by the prescribing physician, who was the only one to know the two different team settings in all details. In fact, the study was performed by the treating physician to check if there were differences between the two settings, so to be able to improve his clinical work.

The TEAM Group included 13 patients that have been treated fulfilling at best the SOSORT criteria for brace management [21]: The answers to the SOSORT questionnaire gave 44 (out of 44) criteria respected (grade, “excellent”). The NOT Group was composed by 25 patients treated following the SOSORT criteria only partially: The answers to the SOSORT questionnaire gave 35 criteria respected (grade, “insufficient”). All the differences were located in the sections “All professionals as a team” (3/8 respected) and in the “Physiotherapists” sections (1/5 respected). In Table 1 all details on the SOSORT criteria and the situations of the two compared treating teams have been reported.

**Table 1:** **Answers to the SOSORT questionnaire (**http:// http://www.scoliosisjournal.com/content/4/1/2/additional **) in the two treating teams considered**

**Questionnaire**	**Answers of subgroups**	
	**TEAM**	**NOT**
**All professionals as a team**	**8/8**	**3/8**
1. Do you work in a multiprofessional team (physician, orthotist and eventually physiotherapist), through continuous exchange of information, team meetings, and verification of braces in front of single patients?	Yes	No*
2. Do you give thorough advice and counselling to each single patient and family each time it is needed?	Yes	No*
3. Do the different professionals in your team give the same, previously agreed messages to patients and families?	Yes	No
4. Do you check each single brace in team (physician, orthotist, and possibly physiotherapist)?	Yes	No
5. Do you follow-up regularly each single brace?	Yes	Yes
6. Do you access the patient’s mood and counsel him and the family at brace delivery and at other follow-ups?	Yes	Yes
7. Do you check each single brace clinically and/or radiographically?	Yes	Yes
8. Do you check the brace and patient compliance regularly and reinforce the usefulness of brace treatment to the patient and his/her family?	Yes	No*
**Medical Doctors**	**17/17**	**17/17**
9. Have you been trained by a previous master (i.e. a physician with at least 5 years of experience in bracing) for at least 2 years?	Yes	Yes
10. Did you have at least 2 years of continuous practice in scoliosis bracing?	Yes	Yes
11. Have you prescribed at least 1 brace per working week (~45 per year) in the last 2 years?	Yes	Yes
12. Have you evaluated at least 4 scoliosis patients per working week (~150 per year) in the last 2 years?	Yes	Yes
13. Do you prescribe each single brace to the constructing orthothist?	Yes	Yes
14. Do you write the details of brace construction (where to push and where to leave space, how to act on the trunk to obtain results on the spine) when not already defined “a priori” with the orthotist?	Yes	Yes
15. Do you prescribe the exact number of hours of brace wearing?	Yes	Yes
16. Are you totally convinced of the brace proposed and committed to the treatment?	Yes	Yes
17. Do you use any ethical mean to increase patient compliance, including thorough explanation of the treatment, aids such as photos, brochures, video, etc.?	Yes	Yes
18. Do you verify accurately if the brace fits properly and fulfils the need of the individual patient?	Yes	Yes
19. Do you check the scoliosis correction in all the three planes (frontal, sagittal and horizontal)?	Yes	Yes
20. Do you check clinically the aesthetic correction?	Yes	Yes
21. Do you maximize brace tolerability (reduce visibility and allow movements and activity of daily life as much as possible for the used technique)?	Yes	Yes
22. Do you check the corrections applied?	Yes	Yes
23. Do you follow-up the braced patients regularly, at least every 3 to 6 months?	Yes	Yes
24. Do you reduce standard intervals according to individual needs (first brace, growth spurt, progressive or atypical curve, poor compliance, request of other team members)?	Yes	Yes
25. Do you take the responsibility to change the brace for a new one as soon as the child grows up or the brace loses efficacy?	Yes	Yes
**Orthotists**	**14/14**	**14/14**
26. Have you been working continuously with a master physician (i.e. a physician fulfilling to recommendation 1 criteria) for at least 2 years?	Yes	Yes
27. Did you have at least 2 years of continuous practice in scoliosis bracing?	Yes	Yes
28. Have you constructed at least 2 braces per working week (~100 per year) in the last 2 years?	Yes	Yes
29. Do you construct each single brace according to physician prescription?	Yes	Yes
30. Do you correct each single brace according to physician indications?	Yes	Yes
31. Do you check the prescription and its details and eventually discuss them with the prescribing physician, if needed, before construction?	Yes	Yes
32. Do you fully execute the agreed prescription?	Yes	Yes
33. Are you totally convinced of the brace proposed and committed to the treatment?	Yes	Yes
34. Do you use any ethical mean to increase patient compliance, including thorough explanation of the treatment, aids such as photos, brochures, video, etc.?	Yes	Yes
35. Do you maximize brace tolerability (reduce visibility and allow movements and activity of daily life as much as possible for the used technique)?	Yes	Yes
36. Do you apply all changes needed and, if necessary, even rebuild the brace without extra-charge for patients?	Yes	Yes
37. Do you suggest to change the brace for a new one as soon as the child grows up or the brace loses efficacy?	Yes	Yes
38. Do you check regularly the brace?	Yes	Yes
39. In front of any problem with the brace, do you refer to the treating physician?	Yes	Yes
**Physiotherapists**	**5/5**	**1/5**
40. Do you check the brace when you evaluate/treat a patient wearing a brace?	Yes	No
41. In front of any problem with the brace, do you refer to the treating physician?	Yes	No
42. In front of any problem with the brace, do you avoid to refer to the patient?	Yes	Yes
43. If you are a member of the treating team, have you been trained to face the problems of compliance, and the needs of explanation by the patient or his/her family?	Yes	No**
44. If you are not a member of the treating team, do you avoid acting autonomously?	Yes	No
**TOTAL**	**44/44 Excellent**	**35/44** Sufficient

Notes. * Even if the answer could theoretically be “yes,” in reality the most correct answer is “no.” In fact, even if the team exists – since all engaged professionals work in the same place under the direction of the same MD - APs do not behave as members of the team and/or do not accept involvement (e.g., it happens that they are against the brace and openly state that opinion to patients and parents). ** In this case, training has been made but not accepted by PTs, whose behavior did not change.

The following differences between the two teams must be considered:

Teamwork: There was a strict collaboration between orthotist and physician, who in both settings worked in exactly the same way. However, in NOT there were only weak connections between physician/orthotist and the APs, while in TEAM the AP served as the main aggregator of the whole team, also involving parents and patients in the therapeutic group;

Setting: TEAM involved patients treated in a private institute, while NOT was an outpatient service of a Rehabilitation Department of the Italian Health National Service (HNS).

### Outcomes and statistics

We preliminarily compared the two groups (TEAM and NOT), and analyzed all the collected data. The normal distribution for all continuous variables was checked (Shapiro-Wilk test) and the parametric test was applied only if verified. We used the ANOVA, t-test and chi square tests according to what was appropriate. Due to the reduced numbers involved in this study, we considered the statistical significance for P < 0.05 but also looked for tendencies when 0.05 < P < 0.1.

In the case–control retrospective study, we set two outcomes before starting data collection: The primary outcome was compliance to bracing. We considered two possibilities: A patient was compliant if either he declared to wear the brace at least 90% of what was required, or if the total wearing time, including the days in which he referred to using the brace less, was at least 90% of the prescription; in the final analysis only the second possibility was used, given the difference of just two patients (6 vs. 8). The secondary pre-planned outcome was “Quality of Life” (QoL), which was considered good if patients had at least 4 points at the SRS-22 score and domains. After the preliminary analysis we added a tertiary “post-hoc” outcome: pain. Patients who declared that pain was one of their main problems were differentiated from those who did not.

To check for confounders, we split the population according to the presence or absence of the analyzed outcomes, and we verified the baseline clinical data and the differences for the other variables included in the same questionnaire. For the three outcomes we calculated the odds ratios (OR) with 95% confidence interval (95CI).

## Results

### Cross-sectional study

The response rates were statistically higher in TEAM than NOT, with 92% versus 48% to the compliance questionnaire and 69% versus 40% for the SRS-22. There was no difference for the general data between TEAM and NOT (Table 2).

**Table 2:** We found no difference between the two groups of the population categorized according to the main risk factor that was studied

	**TEAM**	**NOT**	**P**
Age (years)	15.9 ± 1.6	15.7 ± 1.5	NS
Gender (females)	77%	58%	NS
Disease (idiopathic scoliosis)	84%	58%	NS
Scoliosis at start (Cobb degrees)	25.2 ± 8.8	23.0 ± 14.7	NS
Scoliosis at the study (Cobb degrees)	19.5 ± 9.4	19 ± 3.5	NS
Result obtained (Cobb degrees)	−5.7 ± 4.3	−4.0 ± 6.0	NS
Years of treatment	1.5 ± 0.5	1.2 ± 0.4	NS

TEAM: patients treated by a complete team respecting the SOSORT criteria (score: excellent); NOT: patients treated in a team not respecting the SOSORT criteria (score: insufficient). (NS: Not Significant).

**Figure 1:**
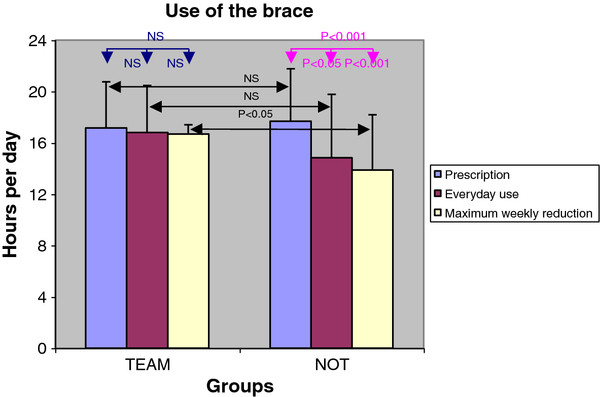
**Use of the brace in the two groups.** There was no statistically significant differences between prescription and everyday usual usage in TEAM, while there was in NOT. Each of the two groups had, once a week, a statistically significant reduction in usage when compared to prescription, but only in controls when compared to the usual usage.

**Figure 2:**
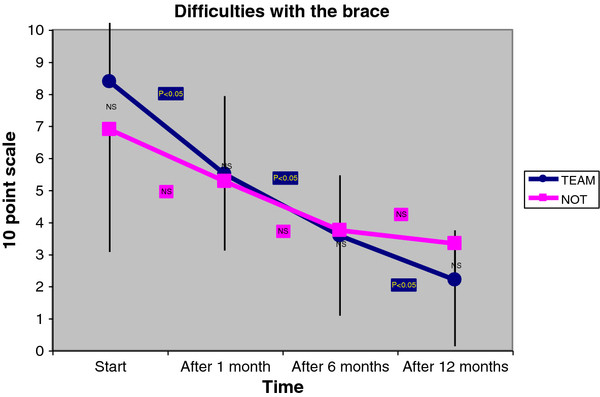
**We found no statistically significant difference in terms of difficulties using the brace between the two groups at each time step.** However, while TEAM improved continuously in a statistically very significant way, in NOT we found a statistically significant improvement only between immediate and 6 months’ difficulties and a statistical tendency in the first month.

TEAM was more complaint to bracing than NOT, and it showed fewer hours in the reduction of brace usage during the week; TEAM also performed nearly twice the minutes of exercises per session than NOT did (Table 3). The self-reduction of bracing hours was statistically significant in NOT only (Figure 1). However, only TEAM showed a significant reduction in the difficulties due to bracing from immediate wearing, to 1, 6 and 12 months (Figure 2). Pain was perceived by 55% of NOT versus 7% of TEAM (p < 0.05) (Table 4). For that reason pain was added to the case–control study as the tertiary outcome. The SRS-22 total score (4.03 ± 0.53 in TEAM vs. 3.53 ± 0.43 in NOT) and the domain “function” (4.13 ± 0.47 in TEAM vs. 3.39 ± 0.60 in NOT) were significantly different between the groups (Table 5). We did not find any difference between the groups neither for how the team was perceived by patients, nor for the difficulties of relationships by patients with the various professionals.

**Table 3:** Treatments prescribed and performed, and compliance according to the main risk factor studied

			**TEAM**	**NOT**	**P**
Bracing	Prescription	hours per day	17.2 ± 3.6	17.7 ± 4.1	NS
	Done		16.8 ± 3.7	14.8 ± 4.9	NS
	Reduction once a week		0.5 ± 0.7	3.8 ± 4.3	<0.05
Exercises	Prescription	session/month	10.5 ± 6.3	13.7 ± 12.6	NS
	Done		7.9 ± 3.7	7.8 ± 4.0	NS
	Done	minutes/session	38.5 ± 12.6	20.0 ± 13.5	<0.05
Compliance	Bracing	%	97 ± 6	80 ± 24	<0.05
	Exercises		69 ± 34	50 ± 39	NS

Average ± standard deviation. TEAM: patients treated by a complete team respecting the SOSORT criteria (score: excellent); NOT: patients treated in a team not respecting the SOSORT criteria (score: insufficient). (NS: Not Significant).

**Table 4:** Problems due to the brace

	**TEAM**	**NOT**	**P**
Pain	7.7%	58.3%	<0.05
Respiration problems	46.2%	33.3%	NS
Problems with friends	7.7%	8.3%	NS
Problems with clothes	23.1%	41.7%	NS
Problems toileting	7.7%	16.7%	NS
**No problem at all**	**15.4%**	**8.3%**	**NS**

TEAM: patients treated by a complete team respecting the SOSORT criteria (score: excellent); NOT: Patients treated in a team not respecting the SOSORT criteria (score: insufficient). (NS: Not Significant).

The prevalence of pain was the only difference between the two populations categorized according to the main risk factor.

**Table 5:** Answers to the SRS-22 total score and single domains

	**TEAM**	**NOT**	**P**
Function	4.13 ± 0.46	3.39 ± 0.60	<0.05
Pain	3.93 ± 0.55	3.54 ± 0.83	NS
Body image	3.86 ± 0.71	3.40 ± 0.66	NS
Mental health	4.13 ± 0.80	3.76 ± 0.84	NS
Satisfaction with treatment	4.16 ± 0.93	3.54 ± 1.08	NS
**Total**	**4.03 ± 0.53**	**3.53 ± 0.43**	**<0.05**

TEAM: patients treated by a complete team respecting the SOSORT criteria (score: excellent); NOT: patients treated in a team not respecting the SOSORT criteria (score: insufficient). (NS: Not Significant).

Average ± standard deviations have been reported for the two groups.

### Case–control retrospective study

**Figure 3:**
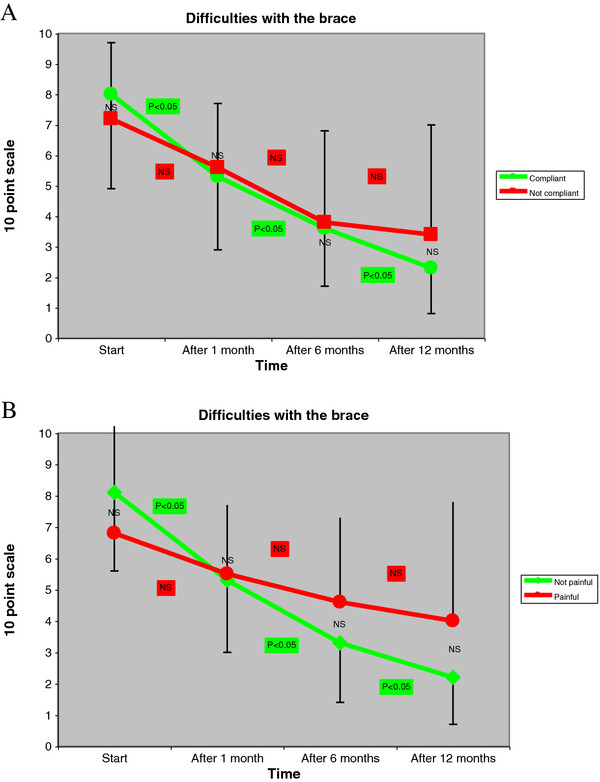
**At the baseline there was no difference for the difficulties due to bracing according to compliance (A) or pain (B).** During treatment the compliant (**A**) and pain-free (**B**) patients improved statistically at each time frame considered, while non-compliant and painful did not. At each time-step there was no difference between the populations. Interestingly, in compliant and pain-free patients, together with the reduction of problems, there was a reduction of the standard deviation of values, so testifying a standardization of the answers. The opposite occurred in non-compliant and painful patients, where the standard deviations increased.

At start of treatment no patient had ever used a brace nor had pain. Unfortunately, the SRS-22 had not been proposed, but we can suppose that, at first evaluation with no previous diagnosis or treatment, QoL was normal and similar between the groups. The problems with the brace were not different for compliant versus non-compliant, nor for painful versus pain-free patients (Figure 3). There were no statistical differences between painful and pain-free patients in terms of compliance (50% vs. 76.5%) and vice versa.

The absence of a good team surrounding the patient increases 5.5 times the risk (OR) of reduced compliance (95CI 3.6-7.4); the ORs of QoL problems and pain were 15.7 (95CI 13.6-17.9) and 16.8 (95CI 14.5-19.1) respectively, with only the Mental Health sub-score of the SRS-22 lacking statistical significance (Table 6).

**Table 6:** Odds ratios and 95% confidence intervals (IC95) of the main outcomes considered in this study

	**Odds Ratio**	**IC95**	**P**
**Primary outcome**			
Compliance with bracing	5.50	3.62-7.38	<0.05
**Secondary outcome**			
Quality of life (SRS-22)	15.75	13.56-17.94	<0.05
· Function	5.33	3.41-7.25	<0.05
· Pain	3.50	1.65-5.35	<0.05
· Body image	8.00	5.56-10.44	<0.05
· Mental health	2.92	0.95-4.89	NS
· Satisfaction with treatment	6.13	4.13-8.12	<0.05
**Tertiary outcome**			
Pain	16.80	14.46-19.14	<0.05

The absence of a complete team is a risk factor for reduced compliance (5.5 times increased risk), quality of life problems apart from Mental Health (range 3.5 to 15.7 times increased risk) and Pain (16 times increased risk). (NS: Not Significant).

## Discussion

This is the first research study that has looked at the role of a complete treatment team, as recently focused in the literature [21], in the management of braced patients. Following an exploratory analysis we performed a case–control retrospective study (first step of prognosis studies) that showed astonishing odds ratios for the studied risk factor (presence or absence of a complete team according to the SOSORT criteria [21]:) 5.5 times increase in non-compliance, 15.7 of QoL disturbances and 16.8 of pain in patients not surrounded and helped by adequate teams.

The immediate answer to bracing clearly shows how this treatment is stressful, with no differences between all the considered groups. Nevertheless, TEAM, compliant and pain-free patients recovered in less time and much better than NOT, non-compliant and painful ones. This means that something helped the first groups more than the second ones. According to the results of this study, the specific help could be neither the physician (prescriptions, treatment management, checking of the brace) nor the orthotist (construction and management of the brace), since they were the same (and behaved in the same way), but it was either the team as a whole or the APs. Consequently, the QoL was dramatically increased, pain decreased and the usage of brace increased. Ultimately we could also expect better results to bracing (due to compliance [13,14]) even if the actual QoL and pain are results “per se.”

As already stated, due to the design the physician and orthotist can be excluded as determining factors of the differences found in the two studied groups. However, when we consider the two different settings as the possible explanation of these results, we should look not only at the organization and collaboration of the team, but also the setting of physiotherapeutic approach (private versus HNS) that could drive certain social differences. Nevertheless, if it is hypothesized that only the wealthiest treat their children in private institutes, it is not necessarily true that the richest are the most compliant with mildly invasive procedures like bracing (it is even possible the contrary); on the other hand, to face at best a stressful event like bracing requires adaptability and elasticity together with external support [23-26]. Even if we found no difference at the baseline for the groups considered, we cannot exclude that people requiring help in a private setting are at the start different from those who seek help from the NHS, and this point should be considered in future prospective studies.

We directed our attention with this study mainly to the team surrounding the patient and particularly on APs. In fact, beyond what they do (i.e., the types of exercises), how they behave and what they say are extremely important. While physicians have the authority of leadership, prescription and indications, while orthotists have the intensity of helping patients in the first impact with the brace, APs play a major role as well, due to their continuous presence at the patient’s side. APs neither prescribe nor build braces, but consequently they appear to the patient as a third, expert judge. Moreover, they have time, due to their continuous weekly work, to explain, sustain, drive and help the patients and their families in a way they can be much more important for the team than the others do. However, they can also be much more destructive: Words like “I would never wear that brace!” or “To me, braces destroy muscles and should not be used” or similar can completely undermine the work of the other members of the team. This is one of the main complaints of the physician and orthotist who participated in this study when considering the APs involved in NOT. This can drive the patients to increased problems and difficulties, as shown in this study, even if they do not perceive this negative impact. In fact, the patients in TEAM and NOT did not perceive their treatment teams differently.

In this study we did not have a group of patients treated in a team in which no AP was involved, and theoretically that should be the real and “pure” control group. Nevertheless, in this situation we would also add the difference due to exercises to that of the team composition. Because we know that exercises do have a favorable effect on scoliosis patients [27,28], and specifically on those braced [29], the actual study is presumably the best way to check for the team role only. Nevertheless, even if we believe this is not true, we cannot ignore that the differences found could be due to a negative effect of the APs in NOT more than to a positive effect of those involved in TEAM.

When we started this study, we mainly hypothesized the possibility that the absence of a good team could incur reduced compliance, while we were not really concerned with QoL issues. Thus we proposed the SRS-22 only for the purposes of checking. Only after the exploratory research did we add pain as a possible outcome. The final results on pain and QoL were very impressive and, in a way, much more important than those on compliance. In fact, as stated by SOSORT experts, QoL and disability are among the main aims of treatment, being more important than Cobb degrees [30]. In that respect compliance should “only” drive better final results in Cobb degrees, which should ultimately correlate with future QoL. On the contrary, the “actual” QoL is always reduced by bracing [1,2], and if the team is able to decrease this reduction it should be very welcome. The same is completely true in regard to pain: Patient management plays a major role in their pain perception, as it is already well known in the literature, mainly for adults, where low back pain is concerned [31,32]. Consequently, the team role appears to be even greater in this study for QoL and pain issues than it is for compliance.

About pain, it is interesting to see that the pain scale in the SRS-22 was not statistically different between the two groups (Table 5), while the difference was noted in the other questionnaire we proposed (Table 4). We must note here that the SRS-22 explore the everyday pain experience mainly related to scoliosis, and not to bracing. Conversely, the questionnaire we developed asked what was the most important problem perceived by patients during brace wearing.

Interestingly, the response rates were different in the two groups. We were very careful in the administration of the questionnaire to guarantee anonymity, with the drawback that it was not possible to check who responded to the questionnaires. Moreover, it was not possible to specifically solicit the answers of non-responders. We consider the reduced response rate as another indication of the greater compliance and better team approach in TEAM than NOT: In fact, responders should be more numerous if patients are highly motivated. Moreover, when we consider that the best patients prefer to show how good they are, an “intent-to-evaluate” analysis, in which we would consider as “failure in compliance” those who did not answer, would only increase the differences found in favor of TEAM. The main limitations we must consider in this study include the following:

Design: A case–control retrospective study allows one to find correlations with possible risk factors but not to draw cause-effect relationships, for which future prospective cohort studies, including a logistic multiple regression analysis, should be planned. Consequently, here we have odds ratios and neither relative nor absolute risks. Nevertheless, in the absence of any study this design is the appropriate first step toward a better understanding.

Selection bias: As discussed above, it is possible that auto-selected patients according to the choice of being treated in a private institute (versus the NHS) are more compliant for other characteristics than the treatment team would be, and this requires other designs to be solved; moreover, in a retrospective study it is not possible to check for patients who abandoned treatment. In any case, we found no differences at the baseline between the groups.

Confounding bias: We controlled for this, but nevertheless we must consider that pain, QoL and compliance could be interrelated and one could drive the others. Only a multiple logistic regression analysis coming from a prospective study will in the future make it possible to deeply check this possibility.

Population: Reduced sample. This is due to the difficulty of finding a population treated in two separate teams while maintaining the same physician, orthotist, and exercises approach. This did not allow sub-analysis to be performed. However, and conversely, due to the reduced statistical power, reaching the statistical significance meant showing solid results.

Population: Various pathologies included. This was necessary in order to have the sufficient numbers needed to reach at least some conclusions. Since we found no difference between the two studied groups for the factor of “disease,” it should not count in the final results. Moreover, we were interested in finding the answers to the stressful event “bracing” in adolescents with spinal deformities instead of a specific disease. According to the clinical experience of clinicians working in TEAM, pain is really rare in braced patients, either for scoliosis or for kyphosis. In fact, the results of this study were surprising for them. As a consequence, results presumably really come from the different settings.

Response rate: Low in NOT. This could interfere with the results even if we explained, as above, the possibility that they could reduce the differences we found.

Compliance measurement: We did not use a compliance meter but instead used only a questionnaire. Nevertheless, we guaranteed complete anonymity to patients, and presumably that allowed us to obtain reliable answers, provided the existing and known “gap” between real and referred use of braces [33].

QoL at baseline. Unfortunately we did not have a QoL measurement at baseline, and consequently we cannot study the differences but only the actual values of the SRS-22 questionnaire.

Conversely, the strengths of this paper include the fact that this is the first study to have looked at the treatment team as a possible factor driving the results: This is a topic that is relatively “hot” in the literature, since it has been underlined only recently [21]. Moreover, as already stated above, the reduced sample considered, with its low statistical power, allows us to state that the differences we found are very strong.

In this respect, it could also be argued that the study population is too small to be representative. It must be considered that we have been highly selective in choosing the population so to respect the inclusion criteria required by the study. In fact, it is very difficult in the everyday clinical life to have a situation where it is possible to explore different teams with the same physician and orthotist involved. Usually physicians work in a single well defined setting, and not in two separate ones like in this specific case. In fact, this study was performed because it was in the interest of the physician to understand if the differences he was seeing between the two team settings were real or not: his aim was to increase the quality of his work in both settings according to the final results. So, when looking at the small population, we can on one side consider the work as preliminary to future studies, on the other very well focused on a specific, quite rare, clinical situation. Another final consideration relates the composition of the population. The overall population before the responses reflects some skew (even if not statistically significant) in the NOT group towards males (36%) and kyphosis (32%) vs (23% and 15%) for TEAM. The different response rates in the two groups (92% in TEAM vs 48% in NOT) could have intensified this skew. Since we know that girls show a higher compliance level to bracing in comparison with boys [34], and that patients with thoracic hyperkyphosis are significantly more symptomatic in all SRS-22 domains [35], it would have been important to know the final gender and disease allocation by group. Unfortunately it was not possible, due to the blind compilation of the questionnaires. Even if we did not find any difference (a part from function) in the SRS questionnaire between the two groups (to be expected if kyphosis males subjects were prevalent), future studies should address these points carefully.

## Conclusions

This study is the first ever published in the literature on a topic such as team work in conservative treatment of scoliosis, that has been considered as one of the most important clinical points by the International Society on conservative management of scoliosis – SOSORT. Due to its limitations, results should be interpreted cautiously, even if the study opens new interesting perspectives.

According to these results, it is possible that, if the team is not working properly, mainly on its allied professionals’ side, there is a great risk of pain and decreased QoL. The same is true in regard to compliance with bracing.

Moreover, this study has shown that the SOSORT management criteria can be important for brace treatment [21].

These results seems to confirm that the management of patients, is sometimes neglected, probably because it is not understood or perceived by the actors in play; nevertheless, it could be a main determinant of final results (through compliance) and/or the immediate QoL and pain of patients.

## Competing interests

SN has a stock of ISICO. 'The other author(s) declare that they have no competing interests'.

## Authors’ contributions

MT: planned the study, supervised all phases of data collection, edited the text. AP: collected all data, drafted a first version of the text, and edited the final one. FT: planned the study, treated patients, edited the text. FZ: contributed to statistical analysis, edited the text. AZ: planned the study, treated patients, edited the text. SN: planned the study, supervised all phases of data collection, contributed to statistical analysis, drafted the text. All authors read and approved the final manuscript.

## Supplementary Material

Additional file 1 **Title of data: Compliance questionnaire.** Description of data: we include the original version of the compliance questionnaire used in this study. The original questionnaire was validated in Italian and German. We add here also a not-validated English translation for the reader: we recommend a validation before any use of the English version. Click here for file
