# Supplementary material for: Team care to cure adolescents with braces (avoiding low quality of life, pain and bad compliance): a case–control retrospective study. 2011 SOSORT Award winner
Source: Scoliosis. 2012 Sep 20;7:17. doi: 10.1186/1748-7161-7-17 (PMC3487862; doi:10.1186/1748-7161-7-17)
Supplement: Additional file 1 — Title of data: Compliance questionnaire. Description of data: we include the original version of the compliance questionnaire used in this study. The original questionnaire was validated in Italian and German. We add here also a not-validated English translation for the reader: we recommend a validation before any use of the English version. [file 1748-7161-7-17-S1.pdf]

ADDITIONAL FILE 1

We present the original version of the compliance questionnaire used in this study. The original questionnaire was validated in Italian and German. We add here also a not-validated English translation for the reader: we recommend a validation before any use of the English version.

QUESTIONARIO SULLA COMPLIANCE

ISTRUZIONI: stiamo valutando quanto abbia influenzato (quanta influenza abbia) la terapia del corsetto nella vita di tutti i giorni, quando sono i momenti difficili e quanto importante per te è/è stato il gruppo medico, fisioterapista e tecnico ortopedico.
Eô necessario che tu risponda in maniera sincera ad ognuna di queste domande. I dati verranno analizzati in maniera anonima. La tua esperienza ci farà riflettere se il nostro lavoro è efficiente (sullôefficienza del nostro lavoro), e su come possiamo migliorarlo per venire incontro a te e ai tuoi futuri compagni portatori di corsetto.

SEZIONE CORSETTO

1. Attualmente quante ore di corsetto ti ha prescritto il medico? é ..

2. Quante ore stai portando mediamente il corsetto al giorno? é ..

3. Ci sono dei giorni in cui è più difficile rispettare le ore di terapia?
   - ☐ Si
   - ☐ No

4. Quanti giorni nella (alla) settimana ti discosti dalla terapia?
   - ☐ 0
   - ☐ 1
   - ☐ 2
   - ☐ 3
   - ☐ 4
   - ☐ 5
   - ☐ 6
   - ☐ 7

5. Di (Per) quanto tempo ti discosti dalla terapia prescritta ogni giorno? é ..

6. Il giorno più critico è (anche più possibilità)?
   - ☐ Lunedì
   - ☐ Martedì
   - ☐ Mercoledì
   - ☐ Giovedì
   - ☐ Venerdì
   - ☐ Sabato
   - ☐ Domenica

7. Descrivi la sensazione che hai provato quando hai indossato il corsetto la prima volta:
   - ☐ Molto sgradevole, ho pensato che non avrei mai portato avanti la terapia.
   - ☐ Sgradevole, mettevo (ho messo) in dubbio di (riuscire ad) indossarlo le ore prescritte.
   - ☐ Tutto sommato pensavo peggio, con un poô di abitudine sarei riuscito/a a metterlo.

8. Quanti giorni ti sono serviti per poterlo indossare le ore prestabilite?
   - ☐ Non sono mai riuscito/a ad indossarlo le ore prestabilite.
   - ☐ Più di 20 giorni.
   - ☐ Da 10 a 20 giorni.
   - ☐ Da 2 a 10 giorni.
   - ☐ Subito dal primo giorno.

9. Quale aspetto ti crea(va) più problemi nellôindossare il corsetto?
   - ☐ Il dolore, il corsetto premeva in maniera insopportabile.
   - ☐ Non mi faceva respirare, mi sentivo soffocare.
   - ☐ Disagio nel farmi vedere dai compagni di classe/amici.
   - ☐ Si vedeva sotto i vestiti.
   - ☐ Non riuscivo ad andare in bagno
   - ☐ Nessuno di questi aspetti, non ho avuto difficoltà.

10. Quanto il corsetto influenza(va) il tuo rapporto con i compagni/amici?
    - ☐ Non ho mai indossato il corsetto fuori casa, mi vergognavo.
    - ☐ Ho selezionato solo poche persone a cui lo ho detto, allontanandomi dagli altri.
    - ☐ Non mi sono fatto problemi, lo ho detto a tutti.

11. Quanto il corsetto influenza la tua vita fuori casa?
    - ☐ Non metto il corsetto fuori casa. A volte non rispetto le ore di terapia.
    - ☐ Esco solo durante le ore di libertà dal corsetto, quindi esco meno.
    - ☐ Esco con il corsetto, mi sento un poô limitato/a ma non rinuncio a niente

12. Quanto il corsetto limita la tua attività sportiva?
    - ☐ A causa del corsetto ho rinunciato alla mia attività sportiva.
    - ☐ Non porto il corsetto durante gli allenamenti e partite, anche se non rispetto le ore di terapia.
    - ☐ Ho parlato con il mio allenatore, riesco a conciliare le ore di libertà con gli allenamenti e le partite rispettando le ore di terapia.

13. Quanto il corsetto condiziona le ore di attività fisica a scuola?
    - ☐ Ho avuto lôesonero, lôinsegnante ha chiesto lôesonero.
    - ☐ Non indosso il corsetto i giorni in cui ho ginnastica e non rispetto le ore di terapia.
    - ☐ Gestisco le ore di libertà togliendolo durante lôora di attività motoria.
    - ☐ Riesco ad indossare il corsetto durante lôora di attività motoria.

SEZIONE ESERCIZI:

1. Qual è la prescrizione della frequenza degli esercizi?   é ..

2. Quante volte esegui gli esercizi alla settimana con il terapista?
   - ☐ 1 volta alla settimana
   - ☐ 2 volte alla settimana
   - ☐ 3 volte alla settimana
   - ☐ 1 volta ogni 2 settimane
   - ☐ 1 volta al mese
   - ☐ Mai

3. Quante volte alla settimana esegui gli esercizi a casa?
   - ☐ 1 volta alla settimana
   - ☐ 2 volte alla settimana
   - ☐ 3 volte alla settimana
   - ☐ 1 volta ogni 2 settimane
   - ☐ 1 volta al mese
   - ☐ Mai

4. Per quanto tempo?
   - ☐ 10 minuti
   - ☐ 20 minuti
   - ☐ 40 minuti
   - ☐ 60 minuti

5. Sei regolare nellòeseguire gli esercizi?
   - ☐ Si
   - ☐ No

6. Se no, quante volte al mese più o meno sgarri?
   - ☐ 1
   - ☐ 2
   - ☐ 3
   - ☐ 4
   - ☐ Scrivi tué .

Il giorno più critico è?

- ☐ Lunedì
- ☐ Martedì
- ☐ Mercoledì
- ☐ Giovedì
- ☐ Venerdì
- ☐ Sabato
- ☐ Domenica

7. Com'è (Qual è) il tuo rapporto con gli esercizi?

- ☐ Li faccio volentieri perché sono importanti.
- ☐ È abbastanza difficile organizzarsi ma sono importanti.
- ☐ Trovo difficilmente il tempo per farli quindi li faccio ogni tanto.
- ☐ Non riesco a trovare il tempo per farli.
- ☐ Non credo nella fisioterapia e negli esercizi e non li svolgo perché ho già il corsetto.
- ☐ Non li faccio mai.

SEZIONE TEAM

1. Quanto ti hanno (ha) aiutato/a il gruppo (medico, fisioterapista e tecnico ortopedico)?
   - ☐ Niente (Per niente), ho gestito da solo/a le difficoltà.
   - ☐ Poco, quando ero in difficoltà loro non servivano a molto.
   - ☐ Solo allôinizio. Poi le difficoltà le ho affrontate (ho affrontato le difficoltà) da solo.
   - ☐ Molto, da sempre le cose che mi dicono mi aiutano e se sono in difficoltà cerco il loro aiuto.

2. In caso di difficoltà/crisi, quando non riuscivi più ad indossare il corsetto, quanto ti ha aiutato il gruppo (medico, fisioterapista e tecnico ortopedico)?
   - ☐ Niente (Per niente), ho rinunciato alla terapia senza chiedere aiuto.
   - ☐ Poco, nonostante quello che mi dicessero (hanno detto) ho rinunciato alla terapia.
   - ☐ Abbastanza, ho dovuto ridurre le ora di terapia, ma mi hanno convinto a tenere duro.
   - ☐ Tanto, sono riuscito/a a superare le crisi anche grazie a loro.
   - ☐ Non ho mai avuto difficoltà.

3. Prova a dare un voto da 1 a 10 al disagio della terapia con corsetto, dove 1 è il disagio nullo e 10 disagio insopportabile.
   - ☐ Appena indossato é é é
   - ☐ Dopo un mese é é é .
   - ☐ Dopo sei mesi é é é .
   - ☐ Dopo un anno é é é .

4. Quali delle seguenti prestazioni ti mettono più a disagio? Dai un voto da 1 a 10, dove 1 corrisponde a disagio nullo e 10 a disagio insopportabile.
   - ☐ Visita medica ....
   - ☐ Fisioterapia é é é
   - ☐ Incontro con il tecnico ortopedico é é é

FRAGEBOGEN UEBER DIE COMPLIANCE

ANLEITUNGEN: wir bewerten wie stark die Therapie mit Korsett das Alltagsleben beeinflusst hat, wann sich schwere Situationen ergeben haben und wie wichtig für dich die Figuren Arzt, Physiotherapeut und orthopedischer Techniker sind.
Es ist wichtig dass du jede Frage aufrichtig beantwortest. Die Daten werden anonym ausgewertet. Deine Erfahrungen helfen uns zu verstehen ob unsere Arbeit effizient ist und was wir verbessern können um dir und deinen zukünftigen Mitpatienten, die auch ein Korsett tragen, entgegen kommen zu können.

KORSETTABSCHNITT

1. Wieviele Stunden hat der Arzt dir verschrieben das Korsett zu tragen? é ..

2. Wieviele Stunden trägt du durchschnittlich das Korsett pro Tag? é é

3. Gibt es Tagen wo es schwieriger ist, die Therapiestunden zu respektieren?
   - ☐ Ja
   - ☐ Nein

4. Wie oft pro Woche respektierst du nicht die Therapie?
   - ☐ 0
   - ☐ 1
   - ☐ 2
   - ☐ 3
   - ☐ 4
   - ☐ 5
   - ☐ 6

5. Wieviel Zeit verbringst du ohne die vergeschriebene Therapie? é é

6. Weches ist der kritischste Tag?
   - ☐ Montag
   - ☐ Dienstag
   - ☐ Mittwoch
   - ☐ Donnerstag
   - ☐ Freitag
   - ☐ Samstag
   - ☐ Sonntag

7. Erklaere den Eindruck, den du gehabt hast, als du fuer das erste Mal das Korsett getragt hast?

- ☐ Sehr unangenehm, ich habe gedacht dass ich die Therapie nicht weiter machen konnte.
- ☐ Unangenehm, ich zweifelte ob ich es alle verschreibenen Stunden tragen konnte.
- ☐ Insgesamt habe ich es mir schlechter vergestellt, ich werde in der Lage sein mit ein bisschen Gewohnheit das Korsett zu tragen.

8. Wieviel Tage hast du gebraucht um das Korsett die richtigen Stunden zu tragen?

- ☐ Ich war nie in der Lage das Kosett die verschrieben Stunden zu tragen.
- ☐ Mehr als 20 Tage.
- ☐ Von 10 bis 20 Tage.
- ☐ Von 2 bis 10 Tage.
- ☐ Seit dem ersten Tag.

9. Welcher Aspekt hat dir Probleme bereitet mit dem Korsettragen?

- ☐ Der Schmerz, das Korsett drueckte und war unertraeglich.
- ☐ Es verbot mir zu atmen, ich erstickte fast.
- ☐ Das Unbehangen, ich wollte nicht, dass meine Klassekameraden mich so sahen.
- ☐ Es war sichtbar unter den Kleidern.
- ☐ Ich war nicht in der Lage aufs Klo zu gehen
- ☐ Nichts von diesen Aspekten, ich hatte keine Schwierigkeiten.

10. Wieviel hat das Korsett dein Verhaeltniss mit deinen Klassenkameraden/Freunden beeinflusst?

- ☐ Ich habe nie das Korsett ausser Hause benutzt, weil ich mich schäme.
- ☐ Ich habe nur einige Leute ausgesucht,und ich habe mich von den anderen entfernt.
- ☐ Ich hatte kein Problem gehabt, ich habe es allen gesagt.

11. Wieviel hat das Korsett dein Leben auswaerts beeinflusst?

- ☐ Ich trage das Korsett nie auswaerts, indem ich die Therapiestunde nicht respektiere.
- ☐ Ich gehe nur waehrend der Korsettfreiheitstunde aus, deswegen gehe ich wenig aus.
- ☐ Ich gehe mit dem Korsett aus, ich fuehle mich ein bischen eingeschraenkt aber ich verzichte auf nichts.

12. Wieviel hat das Korsett deine sportliche Aktivitaet beeinflusst?

- ☐ Ich habe wegen dem Korsett auf meine sportliche Aktivitaet verzichtet.
- ☐ Ich trage nie das Korsett waehrend dem Training und den Wettkaempfen, auch wenn ich dadurch die Therapie Stunden nicht respektiere.
- ☐ Ich habe mit meinem Trainer gesprochen, ich vereinbare die Freiheitstunden mit dem Training und den Wettkaempfen, indem ich die Therapiestunde respektiere.

13. Wieviel hat das Korsett die physikalische Aktivitaet in der Schule beienflusst?

- ☐ Ich war von der Gymnastik befreit.
- ☐ Ich trage das Korsett waehrend der physikalischen Aktivitaet nicht, auch wenn das die Therapiestunden vermindert.
- ☐ Ich teile mir die Freiheitstunden so ein, dass ich waehrend der physikalischen Aktivitaet ausziehe, ohne dabei Therapiestunden zu verlieren.
- ☐ Ich bin ich der Lage während der physikalischen Aktivität das Korsett zu tragen.

ÜBUNGSABSCHNITT

1. Welche Übungsverschriebung hast du? é é

2. Wie oft machst du Physiotherapie?
   - ☐ Einmal pro Woche
   - ☐ Zweimal pro Woche
   - ☐ Dreimal pro Woche
   - ☐ Einmal jede zweite Woche
   - ☐ Einmal pro Monat
   - ☐ Nie

3. Wieoft betreibst du die Übengen zu Hause?
   - ☐ Einmal pro Woche
   - ☐ Zweimal pro Woche
   - ☐ Dreimal pro Woche
   - ☐ Einmal jede zweite Woche
   - ☐ Einmal pro Monat
   - ☐ Nie

4. Für wie lange?
   - ☐ 10 Minuten
   - ☐ 20 Minuten
   - ☐ 40 Minuten
   - ☐ 60 Minuten

5. Machst du die Übungen regelmäßig?
   - ☐ Ja
   - ☐ Nein

6. Wenn nicht, wieoft pro Monat machst du sie nicht?
   - ☐ 1
   - ☐ 2
   - ☐ 3
   - ☐ 4
   - ☐ Schreib dué .

7. Welcher Tag ist der kritischste?

- ☐ Montag
- ☐ Dienstag
- ☐ Mittwoch
- ☐ Donnerstag
- ☐ Freitag
- ☐ Samstag
- ☐ Sonntag

8. Wie ist deine Erfahrung mit den Übungen?

- ☐ Ich mache sie gerne, ich glaube dass sie sehr wichtig sind.
- ☐ Es ist sehr schwierig alles zu organisieren, aber ich schaffe sie, weil sie wichtig sind.
- ☐ Ich finde nicht die Zeit sie zu machen.
- ☐ Ich glaube nicht an die Physiotherapie und ich schaffe sie nicht, weil ich das Korsett trage.
- ☐ Ich mache sie nie.

TEAMABSCHNITT

1. Wieviel hat dir die Gruppe Arzt, Physiotherapeut und orthopedischer Techniker geholfen?

- ☐ Nichts, ich habe allein die Schwierigkeiten bewaeltigt.
- ☐ Nur ein wenig, wenn ich Schwierigkeiten hatte, waren sie nicht so nützlich.
- ☐ Nur am Anfang. Dann habe ich allein die Schwierigkeiten geloest.
- ☐ Viel, immer halfen mir die Informationen, die sie mir gaben. Wenn ich Schwierigkeiten hatte, suche ich ihre Hilfe.

2. Als du Schwierigkeiten/Krisen hattest, als du nicht mehr das Korsett tragen konntest, wieviel hat dir die Gruppe Arzt , Physiotherapeut und orthopedischer Techniker geholfen?

- ☐ Nichts, ich habe auf die Therapie verzichtet ohne Hilfe zu fragen.
- ☐ Nur ein wenig, auch wenn sie mir sagten die Therapie zu machen, habe ich auf sie verzichtert.
- ☐ So so, ich musste die Therapiestunde senken, aber sie haben mich ueberzeugt die Zaehne zusammen zu beissen.
- ☐ Echt viel, ich habe geschafft dank ihnen die Krisen zu bewältigen und jetzt respektiere ich die Therapiestunden.
- ☐ Ich hatte keine Schwierigkeiten.

3. Gib eine Note von 1 bis 10 fuer das Unbehangen der Korsettherapie, wo 1 keine Unbehangen ist und 10 das unertraeglichste Gefuehl ist.

- ☐ Sofort é é é
- ☐ Nach einem Monat é é é .
- ☐ Nach sechs Monate ....
- ☐ Nach einem Jahr é é ...

4. Welche Leistungen geben dir mehr Unbehangen? Gib eine Note von 1 bis 10, wo 1 keine Unbehangen ist und 10 das unertraeglichste Gefuehl ist.

- ☐ Artzlicher Termin ....
- ☐ Physiotherapie é é é
- ☐ Termin mit dem orthopedischen Techniker é é é

COMPLIANCE QUESTIONNAIRE

The original questionnaire was validated in Italian and German. We add here also a not-validated English translation for the reader: we recommend a validation before any use of the English version.

Instructions: we are evaluating the influence of brace therapy on your everyday life, the hard times and the importance for you of the team composed by physician, physiotherapist and orthotist.
We need you to answer sincerely to all questions. All data will be analyzed anonymously. Your experience will help us improving our work for you and for those who will wear a brace in the future.

SECTION ñBRACEò

1. At the present time, how many hours of bracing per day have you been prescribed ? é ..

2. How many hours per day on average are you wearing your brace ? é ..

3. Are there days in which it is more difficult to comply with the hours of therapy?
   - ☐ Yes
   - ☐ No

4. How many days per week do you fail to comply with the hours of therapy ?
   - ☐ 0
   - ☐ 1
   - ☐ 2
   - ☐ 3
   - ☐ 4
   - ☐ 5
   - ☐ 6
   - ☐ 7

5. How many hours per day do you normally fail to comply with the therapy ? é ..

6. Which is the most critical day? (one or more answers)
   - ☐ Monday
   - ☐ Tuesday
   - ☐ Wednesday
   - ☐ Thursday
   - ☐ Friday
   - ☐ Saturday
   - ☐ Sunday

7. Describe your sensation when you wore the brace for the first time:
   - ☐ Very unpleasant, I thought that I would have never been able to follow the treatment.
   - ☐ Unpleasant, I doubted to be able to wear it the number of hours prescribed.
   - ☐ Not so bad as expected: with time I would have been able to use it.

8. How many days did you need before you reached the hours of bracing prescribed?
   - ☐ I've never been able to wear it the hours prescribed.
   - ☐ More than 20 days.
   - ☐ From 10 to 20 days.
   - ☐ From 2 to 10 days.
   - ☐ Since the first day.

9. What is the biggest problem using the brace?
   - ☐ Pain: brace pressure is unbearable.
   - ☐ I cannot breath, I choke.
   - ☐ Discomfort in being seen by classmates/friends.
   - ☐ It can be seen under clothes.
   - ☐ I cannot go to the toilet.
   - ☐ None of these, no difficulties.

10. How much does the brace influence your relationship with classmates/friends?
   - ☐ I've never worn the brace outside of my house because I was ashamed.
   - ☐ I chose few people to whom I told it, and turn from the others.
   - ☐ I thought it was not a big deal, I told everyone.

11. How much does the brace influence your life outside of the house ?
   - ☐ I don't wear the brace outside of my house. Sometimes I don't comply with the hours of therapy.
   - ☐ I only go out when I'm not supposed to wear the brace, so I go out less often.
   - ☐ I go out wearing the brace, I feel a little limited but I don't give up anything.

12. How much does the brace prevent you from practicing physical activity ?
   - ☐ Because of the brace I renounced physical activity.
   - ☐ I don't wear the brace during practice and matches even if I don't comply with the hours of therapy.
   - ☐ I talked to my trainer, I practice when I'm not supposed to wear the brace and I'm able to comply with the hours of therapy.

13. How much does the brace influence the hours of physical education at school?
   - ☐ I was excused from physical education, the teacher asked for the exemption.
   - ☐ I don't wear the brace when I have physical education and I don't comply with the hours of therapy.
   - ☐ I manage my time without the brace so that I can take it off during physical education.
   - ☐ I can keep wearing the brace during physical education.

SECTION ñEXSERCISESò:

8. How frequently are you prescribed to take exercises? é ..

9. How many times per week do you take exercises with your therapist?
   - ☐ 1 time per week
   - ☐ 2 times per week
   - ☐ 3 times per week
   - ☐ 1 time every 2 weeks
   - ☐ 1 time per month
   - ☐ Never

10. How many times per week do you take your exercises at home?
    - ☐ 1 time per week
    - ☐ 2 times per week
    - ☐ 3 times per week
    - ☐ 1 time every 2 weeks
    - ☐ 1 time per month
    - ☐ Never

11. How long?
    - ☐ 10 minutes
    - ☐ 20 minutes
    - ☐ 40 minutes
    - ☐ 60 minutes

12. Do you take your exercises regularly?
    - ☐ Yes
    - ☐ No

13. If you donõt, how many times per month do you fail to take them?
    - ☐ 1
    - ☐ 2
    - ☐ 3
    - ☐ 4
    - ☐ Write your answeré .

14. Which is the most critical day?

- ☐ Monday
- ☐ Tuesday
- ☐ Wednesday
- ☐ Thursday
- ☐ Friday
- ☐ Saturday
- ☐ Sunday

15. How do you feel about exercises?

- ☐ Iôm glad to take them because they are important.
- ☐ Itôs quite difficult to find the time but they are important.
- ☐ Itôs hard for me to find the time so I take them occasionally.
- ☐ I canôt find the time to take them.
- ☐ I donôt believe in physiotherapy and in exercises and I donôt take them because Iôm already wearing the brace.
- ☐ I never take them.

SECTION ñTEAMò

1. How much did the team help you (physician, physiotherapist and orthotist)?
   - ☐ It didnõt help me at all, I faced my problems by myself.
   - ☐ Not much, when I had problems they didnõt help a lot.
   - ☐ Only at the beginning. Then I faced problems by myself.
   - ☐ A lot, what they tell me help me through and if I have problems I look for their help.

2. When you had moments of crisis and you couldnõt wear the brace anymore, how much did the team help you (physician, physiotherapist and orthotist)?
   - ☐ It didnõt help me at all, I renounced the therapy without asking for help.
   - ☐ Not much, in spite of what they told me I renounced the therapy.
   - ☐ Quite a lot, I had to reduce the hours of therapy, but they persuaded me to hold on.
   - ☐ A lot, I managed to go through my crisis also because of them.
   - ☐ Iõve never had crisis.

3. Try to evaluate on a 1 to 10 scale the uneasiness of the brace therapy: 1 means no uneasiness, 10 means unbearable uneasiness.
   - ☐ When you wore it for the first time é é é
   - ☐ After one month é é é .
   - ☐ After six months é é é .
   - ☐ After one year é é é .

4. Which of the following experience makes you feel more uneasy? Evaluate them on a 1 to 10 scale: 1 means no uneasiness, 10 means unbearable uneasiness.
   - ☐ Medical examination ....
   - ☐ Physiotherapy é é é
   - ☐ Meeting with the orthotist é é é
